# Where Do I Fit In? A Perspective on Challenges Faced by Asian American Medical Students

**DOI:** 10.1089/heq.2020.0158

**Published:** 2021-05-19

**Authors:** Daniel J. Ahn, Namrata Garg, Aaditi G. Naik, James Fan, Helen Wei, Bonnie B. Song, Kevin Chung, Monica B. Vela, Karen E. Kim

**Affiliations:** University of Chicago Pritzker School of Medicine, Chicago, Illinois, USA.

**Keywords:** Asian health disparities, Asian American medical students, diversity and inclusion

## Abstract

Asian American medical students (AAMSs) face significant bias in the medical learning environment and are more likely than White students to perceive their school climate negatively. Little is known about the factors that contribute to AAMSs' negative experiences. This perspective aims to describe AAMSs' experiences with diversity and inclusion efforts using survey data from a midwest regional conference, Asians in Medicine: A Conference on Advocacy and Allyship. AAMS respondents reported feeling excluded from diversity and inclusion efforts and conference participants advocated for institutional culture and climate assessments stratified by race and disaggregated into Asian subgroups.

## Introduction

Asian Americans face a unique experience of representing a minority 6.5% of the U.S. population yet constituting 21% of matriculating U.S. medical students.^1^ Therefore, they are not categorized as an underrepresented group in medicine (UiM). However, the literature shows that Asian American medical students (AAMSs), similar to UiM students, perceive their overall medical school learning environment more negatively and report higher levels of stress than White students.^2^ AAMSs face discrimination in the form of harassment, bigotry from attending physicians, and inequitable performance evaluations during clinical rotations.^3–5^ AAMSs are less likely than White students to be inducted into Alpha Omega Alpha honor society even after controlling for the United States Licensing Exam (USMLE) Step 1 scores, research experiences, community service, and leadership opportunities.^6^ AAMSs are significantly less likely than White students to be described with words such as “exceptional,” “best,” or “outstanding” in Medical Student Performance Evaluations.^7^ Other than a single-institution study on how Asian American/Pacific Islander women navigate the medical learning environment,^8^ we also found an overall lack of research on the AAMSs' experiences within the learning environment.

To call attention to these issues and begin exploring solutions, the Asian Pacific American Medical Students Association (APAMSA) and the South Asian Medical Students Association (SAMSA) chapters at the University of Chicago Pritzker School of Medicine hosted the inaugural Asians in Medicine: A Conference on Advocacy and Allyship that took place in 2019. The 1-day conference provided a review of Asian American health disparities and stereotypes about Asian Americans in medicine and emphasized the importance of generating data on Asian American health issues. A community advocacy panel featured leaders from nonprofit organizations serving a diverse range of Asian American populations throughout Chicago. Leaders shared career trajectories, personal challenges, and how their identities as Asian Americans influence their work. Moderated small group discussions focused on (1) building trust with minority patients, (2) reflecting on how identities as Asian Americans inform medical practice and learning how to deal with challenges to identities, and (3) establishing pathways to allyship with other racial minority communities while recognizing Asian American privilege.

Seventy-six undergraduates, medical students, and faculty from six academic medical centers in Chicago attended the conference. Out of the 38 medical students present, 34 were AAMSs (90%), representing five different medical schools in Chicago. A preconference survey with 27 questions was sent electronically 1 week before the conference to all registered individuals. Questions focused on participants' demographics, attitudes about mentorship, as well as support from student groups and institutional diversity programs. A 30-question postconference survey was sent electronically to registered individuals. Questions evaluated conference modules and gauged changes in attitudes about mentorship and institutional support. Both surveys were based on questions adapted from a published focus group discussion guide on racial minority perspectives of academic medicine^9,10^ and the 2018 Medical Student Year Two Questionnaire (Y2Q) from the Association of American Medical Colleges (AAMC).^11^ Google Forms (Google, Mountain View, CA) were used to collect responses.

A total of 36 AAMSs completed the preconference survey, and 15 finished the postconference survey. The majority of participants presurvey were male (58%), attending the University of Chicago Pritzker School of Medicine (61%), and were in their first year of medical school (56%); results were similar postsurvey (Table 1). Preconference, AAMS respondents identified two needs: (1) knowledge on topics related to Asian American health and (2) provision of a safe space where AAMSs can feel confident that they will not be exposed to bias and can learn how to advocate for themselves while supporting other racial minority groups (Table 2). Only one of the six medical schools had required coursework that includes topics related to Asian health disparities, according to respondents. AAMS respondents identified the need for a safe space community that could serve several functions: (1) a place to develop language to help them verbalize challenges they face while simultaneously supporting those faced by other minority groups; (2) a forum for shared backgrounds, experiences, and interests; and (3) a base for AAMSs who are interested in collaborating with other racial affinity groups. The literature supports these viewpoints. Cultural centers have been noted to offer an affirming space for Asian American undergraduates to celebrate their complex cultures and histories, confront stereotyping and racism, combat anxiety and social withdrawal, and seek guidance from professional staff and faculty who are knowledgeable of beneficial campus resources such as student counseling.^12–15^

**Table 1.** Self-Reported Demographics of Asian American Medical Student Conference Attendees, University of Chicago Pritzker School of Medicine, 2019

	Presurvey (*n*=36) *n* (%)	Postsurvey (*n*=15) *n* (%)
Age in years, mean (SD)	24.2 (1.5)^a^	23.8 (1.5)^b^
Gender		
Male	21 (58.3)	10 (66.7)
Female	13 (36.1)	4 (26.7)
Did not respond	2 (5.6)	1 (6.7)
Ethnicity		
Chinese	14 (38.9)	4 (26.7)
Filipino	2 (5.6)	0 (0)
Indian	9 (25.0)	4 (26.7)
Japanese	2 (5.6)	1 (6.7)
Korean	4 (11.1)	2 (13.3)
Malaysian	1 (2.8)	0 (0)
Taiwanese	5 (13.9)	4 (26.7)
Vietnamese	1 (2.8)	1 (6.7)
Burmese	1 (2.8)	1 (6.7)
Did not respond	2 (5.6)	1 (6.7)
Medical school		
University of Chicago Pritzker School of Medicine	22 (61.1)	10 (66.7)
University of Illinois College of Medicine	5 (13.9)	2 (13.3)
Rush Medical College	4 (11.1)	0 (0)
Northwestern University Feinberg School of Medicine	3 (8.3)	2 (13.3)
Loyola University Chicago Stritch School of Medicine	1 (2.8)	1 (6.7)
Year in medical school		
First year	20 (55.6)	11 (73.3)
Second year	7 (19.4)	3 (20.0)
Third year	2 (5.6)	0 (0)
Fourth year	5 (13.9)	1 (6.7)
Gap year	2 (5.6)	0 (0)

^a^ *n*=34.

^b^ *n*=14.

SD, standard deviation.

**Table 2.** Concerns Identified from 36 Pre-Conference Survey Responses by Asian American Medical Students, University of Chicago Pritzker School of Medicine, 2019

Need	Representative quotes from survey responses
More knowledge on Asian American health disparities and culturally competent care	“[I want] to educate myself on Asian American health disparities to better serve future Asian American patients.” “I want to better understand how my identity as a South Asian American will affect my interactions in the clinic and learn how I can help reduce the disparities faced in other minority communities.” “I hope to learn how to provide culturally competent care to Asian and minority patients. I also wish to know what health problems face the Asian American community that could be better addressed by physicians and the healthcare system.”
Safe spaces for AAMSs	“[I am] eager to have a space to talk about identity and hear other people's stories. I want to hear how other people vocalize their feelings.” “I wanted to meet with other Asian Americans in medicine and see how their identities are informing their approach to medicine…” “[I] would love to be a part of a community that is active in being a pillar for change and social justice.”
Perceived exclusion from campus conversations about diversity and inclusion	“I want to feel more confident that my identity as an Asian American deserves to be included in talks about diversity.” “I want [to learn] successful strategies for Asian Americans to participate in and support conversations about diversity/inclusion with other minority groups while also demonstrating that the concerns our community have also belong…” “My experiences with Asian-American culture in general have been ones in which we are encouraged to keep quiet about the problems we face…”

AAMSs, Asian American medical students.

In addition, many AAMSs reported feeling left out of campus conversations about diversity and inclusion (Table 2). This theme is also found in the extant literature—perceived exclusion from inclusion efforts and pressures to remain silent about discrimination are two key factors that have been found to increase stress and anxiety as well as negatively affect job performance in health care organizations.^16^ AAMS respondents described frustration with model minority stereotypes, explaining that the struggles Asian Americans face are often devalued. AAMSs lacked confidence about their racial or ethnic identity and felt obligated to prove that they belong in discussions about race in medicine. Others indicated they often feel unsure about whether microaggressions from classmates or faculty are valid because they are Asian American. This theme of exclusion aligns with research showing that Asian American professionals feel that genuine problems they face are downplayed or ignored due to claims that Asian Americans are a model minority and benefit from reverse discrimination.^17,18^ Faculty and community organizer speakers as well as students from underrepresented Asian ethnic groups advocated for the need to disaggregate AAMSs. “Asian American” is itself a broad term that encompasses >20 different ethnic groups, all with widely varying experiences, historic roots, and challenges.

Postconference, most of attendees responded that they felt empowered about their identity and felt more capable of advocating for themselves, and more confident in requesting education on topics pertaining to Asian American health at their respective schools (Table 3). Our own school established new initiatives. First, the time allotted for instruction on Asian health in the required quarter-long course on health disparities^19^ for first-year medical students has tripled from 1 to 3 h. APAMSA and SAMSA students now speak at weekly interview day breakfasts with applicants about their experiences at Pritzker. Furthermore, the Identity and Inclusion (i2i) Steering Committee,^20^ a student- and faculty-led group that develops programming pertinent to minority groups, hosted us at a general meeting to share our findings from the conference. The medical school also hosted programming for AAMSs and internal medicine residents to discuss negative experiences, such as microaggressions from patients and attending physicians, and how our identity has influenced our calling to medicine. Feedback from this event was overwhelmingly positive; attendees felt recognized as a group that face their own set of struggles and appreciated the opportunity to build a safe space community.

**Table 3.** Theme of Increased Empowerment Identified from 15 Postconference Survey Responses by Asian American Medical Students, University of Chicago Pritzker School of Medicine, 2019

Theme	Representative quotes from survey responses
Increased empowerment about Asian American identity	“I will use the dialogue we had to self-reflect in times when I'm not sure how to interpret another person's actions that are borderline microaggressions. It was helpful to hear the internal dialogue of my peers in these situations, and to feel comforted by the fact that other people go through the same internal conflict as me…” “I feel more comfortable advocating for specific issues that my Asian American community is affected by.” “I want to join the Asian American association at my medical school and become involved in setting up another conference about Asian American health disparities.”

## Conclusion

The support from our medical school has helped us as Asian Americans feel more visible and included in diversity and inclusion efforts. Similar collaborations between AAMSs affinity groups and diversity and inclusion offices may successfully yield a baseline understanding of the needs of AAMSs as well as a feasible agenda to address them and improve AAMSs' experiences at other schools. We offer two recommendations on how this work can be executed: (1) institutional culture and climate assessments that allow for stratification by different identities, especially race, and (2) disaggregation of data on AAMSs.

Medical institutions, specifically their diversity and inclusion offices, should include questions about identity in annual climate and culture assessments to allow for targeted interventions that adequately address the concerns of specific groups of students.^21^ This can help AAMSs feel increasingly valued and visible, ultimately helping them become better physicians.^22^ Medical schools should disaggregate data when examining the experiences and needs of AAMSs. Asian Americans, when separated by ethnic groups, have significant disparities in socioeconomic status, education level, family migration history, and cultural background^23^ that can substantially influence the experiences and perceived challenges of different AAMSs in the medical learning environment. For example, Native Hawaiians and other Pacific Islanders, who are often placed under the label “Asian,” face historical and social barriers that make accessing educational and professional opportunities difficult.^24^ Furthermore, Southeast Asian Americans, such as Cambodian and Laotian Americans, have much lower household incomes and college education completion rates than many South and East Asian Americans.^23^ AAMC data demonstrate that Southeast Asian Americans apply to medical school at a significantly lower rate than East and South Asian Americans.^25^ As a result, without disaggregation, researchers and institutions risk gathering broad perspectives representative of only majority Asian American groups, and the lack of plurality in Asian American voices can result in an oversimplification of the AAMS experience.

AAMSs constitute a significant portion of U.S. medical schools and come from incredibly diverse settings. Identifying the needs of AAMSs and involving them in diversity and inclusion efforts can make AAMSs feel more visible and empower AAMSs to become powerful advocates and allies in efforts to establish a diverse physician workforce. Medical schools can and should improve the learning environment of students who identify as Asian American.

## Abbreviations Used

AAMC: Association of American Medical Colleges
AAMSs: Asian American medical students
APAMSA: Asian Pacific American Medical Students Association
BNGAP: Building the Next Generation of Academic Physicians
SAMSA: South Asian Medical Students Association
SD: standard deviation
USMLE: United States Licensing Exam
